# Health Management of Working Pregnant Nurses: A grounded theory study

**DOI:** 10.1002/nop2.2158

**Published:** 2024-04-19

**Authors:** Marie Hino, Risa Takashima, Rika Yano

**Affiliations:** ^1^ Graduate School of Health Sciences Hokkaido University Hokkaido Japan; ^2^ Faculty of Health Sciences Hokkaido University Hokkaido Japan

**Keywords:** grounded theory, health management, maternal health, nurses, pregnancy

## Abstract

**Aim:**

To explore the recognition of pregnant nurses on how they managed their health conditions to examine safe working strategies.

**Design:**

A qualitative study with a grounded theory approach.

**Methods:**

Twenty‐one nurses engaged in work during their pregnancy were recruited and interviewed using a semi‐structured questionnaire from January to June 2021. The data were analysed using a constant comparative method.

**Results:**

The core category ‘duelling roles’ and the four other categories emerged. Pregnant nurses understand the ‘weight of one’ of being a professional in the workplace. Therefore, despite their health concerns, they struggle to complete their work as one team member to avoid inconveniencing others. However, through experiencing various nursing situations, they ‘perceive one's limits’ of working as they had done before pregnancy and protect their health and patients. Nevertheless, interactions with patients and their colleagues bring ‘delight in nursing’, which encourages them to continue working. Pregnant nurses thus develop a ‘prioritizing the foetus’ working style to continue being nurses while protecting their health.

**Implications for the Profession and/or Patient Care:**

These results provide meaningful guidance in considering safe job retention strategies for pregnant nurses. Sharing and developing the ‘prioritizing the foetus’ mindset and management skills gained by the participants may be beneficial for the appropriate health management of pregnant nurses. The study may also facilitate nursing managers' understanding of the experiences of pregnant nurses and encourage them to consider reviewing nursing practices.

**Reporting Method:**

The Consolidated Criteria for Reporting Qualitative Studies checklist was used to ensure the quality of research reporting.

**Patient or Public Contribution:**

Members of the nursing team were involved in the design, conduct and interpretation of the data in this study.

## 1 INTRODUCTION

Nurses are the largest group of healthcare professionals. Approximately 94% of nurses are women, of whom about 70% are of reproductive age (Organization for Economic Co‐Operation and Development, 2019). However, nurses are found to have an increased risk of perinatal complications (Liao et al., 2019). Some systematic reviews and meta‐analyses have demonstrated that occupational activities and hazards among nurses (e.g., shift work, prolonged standing, physical workload and chemotherapy agents) are associated with several adverse pregnancy outcomes, including miscarriage, congenital malformation, preterm delivery and gestational hypertension (Cai et al., 2019, 2020). To safeguard pregnant workers, various policies are enforced in each country. In Canada, for example, pregnant workers are entitled to job modifications and employers must accommodate those requests (Government of Canada, 2022). In Norway, regulations on reduced working hours and the right to take leave during pregnancy have been established (NAV, 2023). However, not all pregnant workers can easily exercise their rights in some countries. In Japan, only 49.9% of pregnant nurses are exempted from night shifts and 13.1% from hard labour, mainly due to a lack of manpower (Japan Federation of Medical Worker's Unions, 2021). These societal issues require hospital organizational and administrative reforms, but solutions remain elusive.

## 2 BACKGROUND

Health management taken by oneself is one way of safeguarding pregnant workers. Health management, which is easily implemented according to the will and knowledge of the individual, reduces the incidence of perinatal abnormalities (Rezaeean et al., 2020). In particular, pregnant nurses at risk should consciously manage their health (LeVeck, 2021). However, there have been no suggestions on strategies to be adopted in this regard.

Previous studies on work experiences among pregnant nurses have shown that they perceived their work environment as very risky and had health fears. Furthermore, it was not easy for them to avoid their high‐risk duties. The results showed that no matter how painful their pregnancy‐related symptoms were and no matter how concerned they were about their health, they ignored the pain and continued to work (Lee et al., 2020; Rainbow et al., 2021). Quinn (2016) explored how primiparous nurses working in acute care hospitals integrated pregnancy and full‐time employment. The study showed that pregnant nurses attempted to balance their professional challenges and relationships in their work environments following the process of ‘becoming someone different’ from their pre‐pregnancy selves through social interactions. However, in reality, the participants had difficulty balancing them until maternity leave.

Despite these difficulties, there have been no studies to explore the experiences of pregnant nurses in managing their health. Previous studies have only emphasized the need to promote understanding among managers and to revise maternal and foetal protection regulations, but not their health management. To examine measures for pregnant nurses to continue working safely, it is necessary to explore and learn from how pregnant nurses recognize their health management while working.

Therefore, this study explored the recognition of pregnant nurses, regardless of parity, gestational abnormality and ward type, on how they managed their health conditions while working. It suggests strategies to manage their health conditions so that they can continue to work safely.

## 3 METHODS

### 3.1 Design

Corbin and Strauss's grounded theory approach was used in this study. Constant comparative analysis and theoretical sampling in grounded theory set it apart from a purely descriptive analysis, and combining them enhanced the level of data analysis (Chun Tie et al., 2019). The reasons for selecting this method were as follows. First, as this approach is recommended while investigating social situations to which people have to adapt, it was considered appropriate for this study (Corbin & Strauss, 2014). Second, the participants in this study were pregnant nurses. They experienced interactions with the foetuses, colleagues and patients while undergoing dynamic physical and mental changes during pregnancy. Therefore, this approach, which is rooted in the applied methodological area of symbolic interactionism and used for understanding the social process of experience (Corbin & Strauss, 2014), was considered appropriate for this study.

### 3.2 Sampling and recruitment

The study participants were pregnant women or postpartum mothers who were nurses associated with hospitals during pregnancy, between the second trimester (after 28 weeks of pregnancy) and under 1 year postpartum. The exclusion criteria were: (1) being less than 20 years of age, (2) having a history of mental illness and (3) being diagnosed with a significant physical or mental burden. People under the age of 20 were excluded because of the ethical considerations of minors and the physical and psychosocial issues specific to young pregnant women that may distort the results we wanted to explore. While purposive sampling was initially adopted, theoretical sampling was later used based on emerging statements and concepts. There was no relationship between the researchers and participants before the study. The first author provided the following information to potential participants via email: qualifications, institutional affiliation, clinical experience of the researcher group, research objectives, interview methods and ethical considerations. A total of 21 nurses were introduced to the study, and all of them provided consent.

### 3.3 Data collection

Data were collected from participants in Japan from January to June 2021. One‐on‐one semi‐structured interviews were conducted with complete privacy at the researchers' and participants' respective homes using an online platform (Zoom). The interview duration was 32–51 min for each participant. The interview guide was used, and participants' demographic characteristics, such as age, number of pregnancies, years of nursing experience, department, medical history and family structure, were asked at the beginning of the interview. The interview guide's first version was developed by reviewing the literature, and pilot tests were conducted with five women. Based on the results, the content was revised to enable analysis according to the research objectives. These pilot testers were not included in the sample for this study. The final version of the interview guide was used in this study (Table 1). Only one interview with the guide was conducted with each participant. All interviews were conducted by the first author and recorded on an integrated circuit recorder. Participants' facial expressions and moods, as well as the interviewer's emotions, were recorded in field notes. All interviews were conducted in Japanese.

**TABLE 1:** Interview guide.

Main questions	When did you announce your pregnancy? Why?
	2 What and when was the first physical change you felt after pregnancy?
	3 How did your body change as the weeks went by?
	4 How did you manage your maternal‐foetal health during pregnancy? Please describe in detail how you managed each stage of pregnancy.
	5 How did you manage your work during pregnancy? Please describe in detail the management of each stage of your pregnancy.
	6 What were some of the difficulties you faced at work?
	7 Did you have any worries or problems in terms of managing your health and work?
	8 What were your feelings about continuing to work during pregnancy?
Question for multiparas	Compared to your first pregnancy, did your awareness or behaviour change during your second and subsequent pregnancies?
Question for primiparas	How did you think about the priorities of the foetus and your work?
Further clarifying questions	Please describe it in as much detail as possible based on your experiences…
	Please describe how you felt…
	Could you give an example…
	Did you mean…

### 3.4 Data analysis

The data were analysed using a hierarchical, systematic approach to grounded theory (Corbin & Strauss, 2014). First, as part of the open coding process, the first author created an accurate, verbatim transcript by repeatedly playing the interview recordings. Subsequently, the transcript was decomposed to ensure that the meaning of the participants' narratives was not compromised, and codes were created using Microsoft Office Word. The properties and dimensions were extracted from the codes, and labels were given to each code. Then, the labels were compared and categorized based on similarities and differences. The labelling and categorizing process was conducted independently by the three researchers. Subsequently, the coding results were integrated after consultation within the group. In the axial coding process, the second step, the categories were analysed by repeatedly returning to the data and dividing them into phenomena. We also examined the associations between categories for every participant. This process was conducted in the research group, which enabled all event variations to be ascertained.

To collect and analyse the data simultaneously, the processes of open coding and axial coding were repeated at the end of each interview, and theoretical sampling was conducted. In particular, at the end of the seventeenth interview, we found differences between multiparas and primiparas in the category ‘prioritizing the foetus’. Therefore, after the eighteenth interview, primiparas were emphatically asked about their prioritization of the foetus, and multiparas were further asked about the differences in their experiences with their first and second pregnancies. It allowed us to explore this category in more depth. In the selective coding process, the final step, all the categories were integrated into a conceptual framework at a higher abstraction level by conducting repeated examinations. After the twentieth interview, no new categories/subcategories could be found; therefore, we discussed and confirmed having reached theoretical saturation. Finally, a core category emerged from the final category consolidation.

Additionally, field notes were used throughout the processes of labelling, categorizing, examining the relevance of the categories and constructing the theory. The notes provided information about the participants and the interests of the interviewer, which helped all researchers to interpret the participants' statements in depth and in context.

### 3.5 Research team and reflexivity

The first author was a registered nurse, midwife and public health nurse with 8 years of clinical experience. This situation could have affected the results of the study in the following ways. Positively, the participants could have been willing to describe their experiences in an optimistic and professional sphere because the researcher belonged to the same gender and profession as them. However, because the researcher was a nursing professional, she could have stereotypes about the profession (e.g., ‘nurses are patient‐centred’ or ‘midwives have a good knowledge about perinatal care’). She may, therefore, interpret participants' descriptions according to her own ideas and expectations and overlook important aspects of their meanings. To address this issue, the second author, who was a qualified Ph.D., occupational therapist, senior lecturer, researcher in the field of rehabilitation science and expert in qualitative research, was selected. Moreover, the first and second authors had no experience of working as nurses during pregnancy. As such, they could understand the participants' experiences without preconceptions or biases. However, it was also potentially difficult for them to apprehend the participants' experiences in depth. Therefore, the third author, who was a qualified Ph.D., registered nurse, public health nurse, professor, researcher in nursing and had experience working as a nurse during pregnancy, was selected. All the authors were women. The second and third authors assisted in developing the interview guide, coding process and repeated theoretical comparisons and reviews of the data analysis and summary of results and discussion.

### 3.6 Rigour

Data reliability was established following the approach of Lincoln and Guba (1985). To confirm the data credibility, three researchers, including an expert in qualitative research, conducted and evaluated the data analysis, interpreted the results and repeated expert checking of the results. The results and interpretations were given as feedback to the three participants, and a consensus was reached through member checking. After developing the theory, the researchers contacted the three participants again and conducted one interview for each feedback (i.e., the researchers and the three participants had two contacts in total). The timing was determined according to Glaser (1978), who emphasized the significance of avoiding external influences during analysis. To ensure the possibility of transferability, sufficient descriptive data on the researchers and participants were provided in the methods and results sections. The discussion also examines the Japanese nursing work environment in which the study participants work. Regarding dependability, the three researchers repeatedly analysed the interview transcripts, evaluated and reviewed the extracted content, categories and their characteristics and ensured that discussions were held until a consensus was reached among the researchers. Confirmability was accomplished by maintaining an audit trail of all raw data, field notes, data analysis and integration artefacts and related literature. The Consolidated Criteria for Reporting Qualitative Studies checklist was used to ensure the quality of research reporting (Tong et al., 2007).

### 3.7 Ethical considerations

The ethics committee of the institution to which the authors belonged approved this study (No: 20–54, Date: 06.01.2021). Potential participants were provided verbal and written information about the study's purpose, data confidentiality, free will participation, the right to withdraw from research participation, the protection of personal information, strict data handling and recording with a voice recorder. All the participants provided written consent. Additionally, code numbers were used to protect their identity (e.g., Participant 1: No. 1).

## 4 RESULTS

Data were obtained from 14 hospitals and 17 wards across nine cities in Japan. The participants' mean age (standard deviation) was 30.3 (2.1) years and ranged from 27 to 36 years; of the 21 participants, 10 were multiparas and 11 were primiparas. Three had experienced an imminent miscarriage (i.e., vaginal bleeding before 20 gestational weeks) and eight had experienced an imminent preterm birth (i.e., imminent birth before 37 gestational weeks) in this pregnancy. Six had also experienced a previous miscarriage. The participants' characteristics are summarized in Table 2.

**TABLE 2:** The characteristics of the participants.

No	Age (years)	Number of births (miscarriages)	Clinical experience (years)	Diagnosis		Unit	Shift type
				Imminent miscarriages	Imminent pre‐birth		
1	33	1 (1)	8	〇	〇	ICU	Shift
2	33	2 (1)	10	〇	‐	Haematology medicine	Shift
3	30	1 (2)	8	‐	‐	Operating room	Day
4	30	1 (0)	7	‐	‐	ICU	Shift
5	36	1 (0)	12	‐	〇	Mixed ward	Shift
6	32	1 (0)	10	‐	〇	Obstetrics and Gynaecology	Day
7	30	1 (0)	6	‐	‐	Paediatrics outpatient	Day
8	27	1 (0)	6	‐	‐	Mixed ward	Shift
9	30	1 (0)	8	‐	‐	Obstetrics and Gynaecology	Day
10	30	1 (0)	8	‐	‐	Gastroenterology	Shift
11	30	0 (1)	8	‐	‐	Cardiovascular surgery	Shift
12	27	0 (1)	7	‐	‐	Cardiovascular medicine	Shift
13	28	0 (1)	8	‐	‐	ICU	Shift
14	28	0 (0)	6	‐	〇	Nephrology and Rheumatology	Shift
15	32	0 (0)	10	‐	‐	Obstetrics and Gynaecology	Day
16	32	0 (0)	10	‐	〇	Mixed ward	Shift
17	30	0 (0)	7	‐	〇	Obstetrics and Gynaecology	Shift
18	30	0 (0)	7	‐	〇	Cardiovascular medicine	Shift
19	29	0 (0)	7	〇	‐	Mixed ward	Shift
20	30	0 (0)	8	‐	〇	Neurology surgery	Shift
21	30	0 (0)	7	‐	‐	Mixed ward	Shift

Abbreviations: “‐”, no; “〇”, yes; ICU, intensive care unit; pre‐birth, preterm birth; shift, shift work (i.e., rotating shifts, which include day, evening, night or weekend shifts).

**FIGURE 1:**
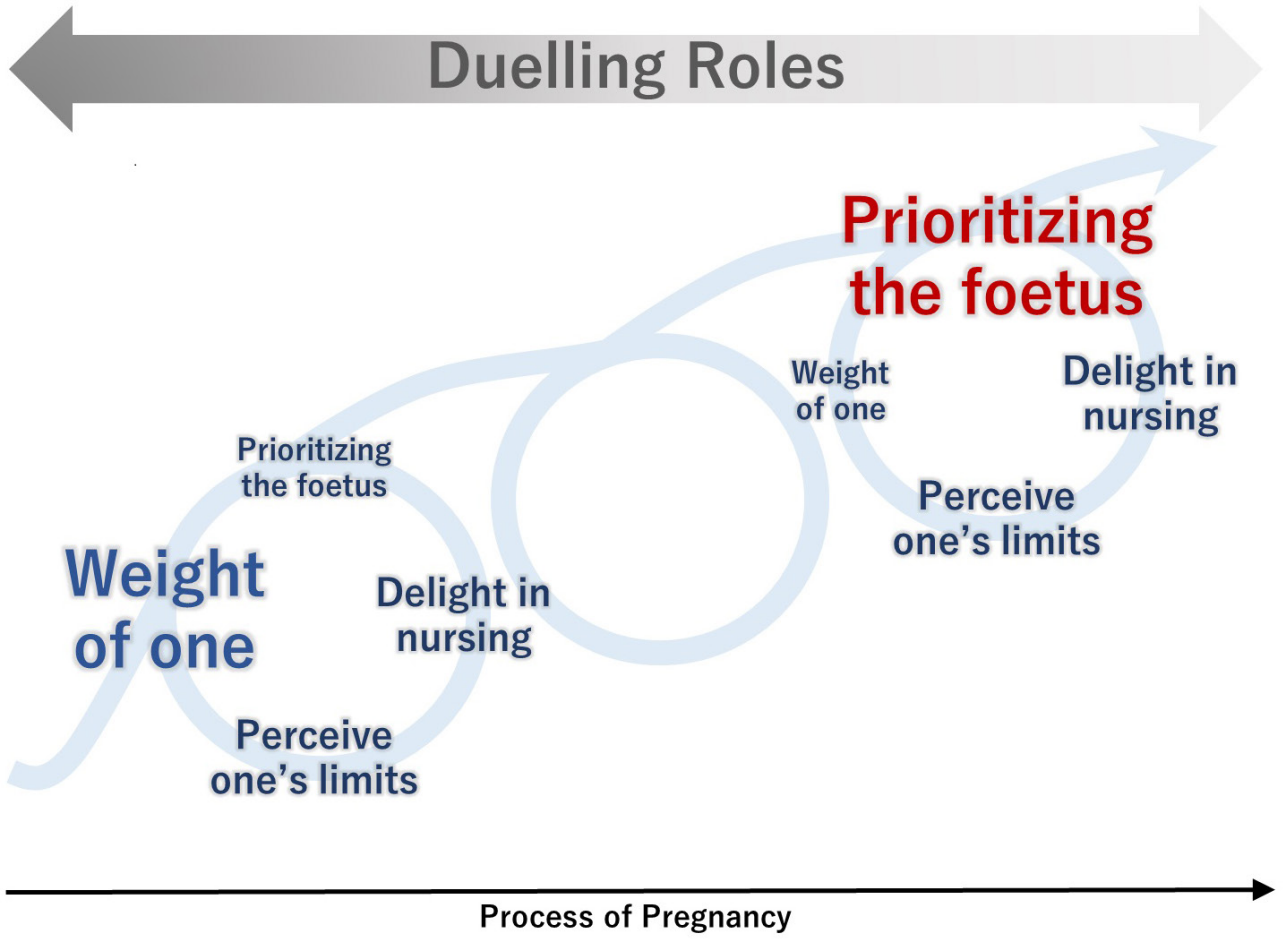
Model of duelling roles among pregnant nurses.

A model was developed to describe the health management process of pregnant nurses (Figure 1), and the core category ‘duelling roles’ emerged. Nurses fully understand the ‘weight of one’ of being professionals in the workplace. Therefore, despite their health concerns, they struggle to complete their work as one team member to avoid inconveniencing colleagues and patients. However, through experiencing various nursing situations, they ‘perceive one's limits’. They feel inadequate or frustrated about losing nursing abilities due to pregnancy and their limited ability to protect their health and patients. They feel the ‘weight of one’, but face the conflict of inability to accomplish the role of one. Nevertheless, interactions with patients and their colleagues bring ‘delight in nursing’, which buffers the conflict and encourages them to continue working. Pregnant nurses seek ways to continue on their job while protecting their health, and come up with a ‘prioritizing the foetus’ working style. They attempt to complete the role to the greatest possible extent while prioritizing foetal life, which only they can protect. Furthermore, pregnancy involves dynamic physical and mental changes, and the hardships and challenges of the time are not constant. Pregnant nurses, therefore, progress through the process of pregnancy, constantly moving back and forth between the four categories. In the ‘duelling roles’ of nurse and pregnant woman, they evolve from ‘a nurse’ to ‘a pregnant nurse’. The four categories describing the process of health management of pregnant nurses are detailed below.

### 4.1 Weight of one

Nurses fully understand the weight of one nurse, which is the pressure to accomplish their responsibilities as part of the team in a busy and understaffed environment, as well as the pressure of being a nurse in the eyes of patients and their families. A nurse who worked shifts at a nephrology and rheumatology department described this as follows:

I was always very worried about my baby. (…) But I felt sorry for the inconvenience to others, I couldn't take a day off, no matter how sick I was…because I understood the weight of one nurse. (…) We feel responsibility in various aspects, including myself as a member of a team and myself as a professional seen by patients. Even if I have a precious child in my belly, it is difficult to put myself first and neglect others. (No. 14)

Another nurse who worked in the intensive care unit said,

So busy, we can't even take a short break. When a life‐and‐death emergency comes in, even a pregnant woman has to be mobilized as a member of the team. It takes five to six years to develop a full‐fledged nurse, and if I were to leave, operations would cease to function (…) I had no option of taking time off because I know the weight of one nurse. (No. 1)

Thus, because pregnant nurses are concerned about inconveniencing their colleagues and patients, they try to somehow complete their one‐person role even if their symptoms are painful.

A participant described that she could not even announce her pregnancy as she felt sorry for her colleagues.

I couldn't announce my pregnancy because I felt sorry for others… While not announcing, I couldn't avoid doing hard work and caring for patients with infections… I had to endure it. (…) Looking back, I even feel that delivering the baby safely was a miracle. (No. 19)

Thus, the harsh working environment made the participants emphasize the ‘weight of one’ repeatedly. Moreover, they felt the ‘weight of one’ throughout the process of working, although to varying degrees.

### 4.2 Perceive one's limits

The physical and mental strain of pregnancy is unexpected for pregnant nurses. A primiparous nurse, who experienced an imminent premature birth during this pregnancy, vividly described the condition:

The body changes during pregnancy were more painful than I imagined. Side effects of tocolytic agents, heart palpitations, hands shaking, and fatigue, tormented me… My big belly also made it impossible to approach the patients and give them adequate care. This caused discomfort and distress to patients. That was both apologetic and painful. (No. 17)

Pregnant nurses thus feel that their nursing abilities are disrupted by the physical changes and burdens of pregnancy. They are unable to provide the same levels of patient care as they did before pregnancy and feel inadequate or frustrated with themselves and sorry for others.

Furthermore, losing nursing abilities due to pregnancy leads to the fear that they would not be able to fully protect the lives of their patients. In this regard, a primiparous nurse who had the dangerous experience of performing cardiopulmonary resuscitation (CPR) during pregnancy described the following:

As a mother, I have to protect my foetus. However, I must also protect the lives of patients as a nurse. (…) One day, I had to do CPR on the night shift. Through this experience, I was seriously worried if I could protect the lives of the patients and my foetus using the work style as before pregnancy…. (No. 11)

These words reflected the limitations of working as they had done before pregnancy.

Pregnant nurses are also perceived to be unsure of how to manage their health, as a primiparous nurse explained:

I thought I was a knowledgeable nurse, but I didn't know anything…Is this sick feeling dangerous enough to take time off work or is it a normal physiological phenomenon? (…) I didn't know what to do, so I worked to forget about it…. (No. 18)

Additionally, a nurse described the consequences of continuing to work without knowing in the following words:

I didn't know at what level of uterine contractions I should stop working. There were no guidelines, so I had to make all my own decisions. Consequently, not being able to make an appropriate decision, I ended up having an imminent premature birth…. (No. 20)

Thus, pregnant nurses perceive their uncertainties and limitations in managing their health. For some participants, the focus on work caused them to forget that they were pregnant women. These uncertainties were expressed in detail by primiparas in particular.

### 4.3 Delight in nursing

For pregnant nurses, working during pregnancy is often dangerous and accompanied by health concerns. However, their interactions with colleagues and patients bring them delight and happiness. A primiparous nurse who experienced an imminent miscarriage during this pregnancy, but continued to work, described the situation well:

Patients' “thank you!” made me, “I am going to do my best”. A patient with dementia who had not said a word since hospitalization, smiled and stroked my belly gently, which made me very happy. (…) My interactions with patients and colleagues supported and cheered me up. (No. 19)

Another primiparous nurse who had experienced a miscarriage in the past described:

Having experienced a miscarriage, I knew of course my workplace was dangerous. So, I wondered if I should stop working during this pregnancy. However, I felt lighter and refreshed by talking with my colleagues and patients. I realized that the workplace is also important for me. (No. 17)

Additionally, multiparous nurses described the importance of their work for mothers in childcare during pregnancy.

For me, the time I spent as a nurse at work was precious. It was my first experience of being pregnant while in childcare, so my body and mind were hard. At work, I could forget about childcare and return to myself, because my work was delightful. (No. 3)

Thus, pregnant nurses are able to continue their work because they realize the delight and significance of being nurses.

### 4.4 Prioritizing the foetus

Pregnant nurses continue to seek ways to continue being nurses during pregnancy and develop a ‘prioritizing the foetus’ way of working. A multiparous nurse who had experienced a miscarriage in the past described:

The most important thing is to protect my health by myself. I had a miscarriage in the past, and I learned the significance of prioritizing the foetus. (…) Nurses are very busy; therefore, I could not say that I would not do this or that (work). So, I chose relatively light work while paying attention to my colleagues. (…) Especially in the early stage, I asked colleagues to help. (No. 2)

In these ways, pregnant nurses become oriented to work safely and realize that they are the only ones who can protect the life of the foetus. They also understand that to work with ‘prioritizing the foetus’, the support of those around them is essential. A multiparous nurse who had experienced an imminent preterm birth in the past described:

It was inevitable that my pregnancy would put a burden on my colleagues, and I felt apologetic when they said, “You should take a break!” However, I thought that it would be good to return the favour by working when I would come back. By changing my mind, I could allow myself to rest when not feeling well.

Pregnant nurses feel bad about the burden that their pregnancies place on their colleagues because they understand the ‘weight of one’. However, they are prepared to inconvenience their colleagues to work safely. They then get the idea of returning the favour to their colleagues by working when they return to work after childbirth. This strategy was particularly described by multiparas and primiparas who had previously experienced abnormalities in their pregnancies.

Multiparous nurses, in particular, use what they were unsure of or felt limited in during their first pregnancies as a basis for their health management. They feel that they have a better grasp of their condition than during their first pregnancies. Additionally, they are able to ascertain the appropriate amount of work to prevent abnormalities. A multiparous nurse who experienced imminent preterm birth during her first pregnancy described:

Based on my experience with my first pregnancy, I knew that I would definitely have an imminent preterm birth if I pushed myself too hard this time. So, I was very careful to not do any demanding work from the beginning of pregnancy. (No. 10)

Another multiparous nurse described the limitations she felt in the previous pregnancy connected with ‘prioritizing the foetus’ in the current pregnancy:

During the first pregnancy, I did not focus on the baby at all. Anyway, I put my work first and worked every day, saying “I am sorry, I am sorry, …” to my baby… (…) Looking back on the experience, I regret it a lot. Therefore, this time, I worked with prioritizing the foetus. (No. 9)

Multiparous nurses also described the impact of their first children:

During my first pregnancy, I could not feel the baby in my womb, so I kept working even though my stomach was a little heavy…. But with my second (pregnancy), my first child—my son—was right in front of me. That made me realize the life here (in my womb), and I had to prioritize the new life. (No. 4).

These words indicate that the role of the mother in childcare facilitates a ‘prioritizing the foetus’ working style.

Thus, ‘prioritizing the foetus’ is a valuable strategy to protect the life of the foetus while performing the required professional role as well as possible.

## 5 DISCUSSION

This study explored the recognition of how pregnant nurses managed their health conditions while working. ‘Duelling roles’ emerged as a core category describing the process of pregnant nurses' health management. The roles of a nurse feeling ‘weight of one’ and a pregnant woman wanting to ‘prioritizing the foetus’ are relative, and the duelling roles continue unless she takes maternity leave. However, through various interactions, they develop a ‘prioritizing the foetus’ way of working while perceiving their limitations and feeling delight in nursing. They then gradually increase the weight of ‘prioritizing the foetus’ and evolve as pregnant nurses.

Previous studies reported that pregnant nurses had a disconnect between wanting to protect their health and completing nursing tasks. For example, Rainbow et al. (2021) showed that pregnant nurses worked hard in the desire to be ‘super nurses’, carrying out patient care instead of caring for themselves. Quinn (2016) also found that pregnant nurses worked to be considered ‘good nurses’ by colleagues. However, we recently found that the concept of the ‘weight of one’ affected the struggles of pregnant nurses.

The ‘weight of one’ could be related to the working environment of nurses in Japan. For example, the number of nurses per 100 beds is far lower in Japan, that is, 87.1 compared to 427.6 in the United States (Organization for Economic Co‐Operation and Development, 2019). Additionally, the number of patients per nurse is more than seven, and on the night shift, more than 10 in Japan, compared to a maximum of five in California, the United States (Japan Federation of Medical Worker's Unions, 2021; United Nurses Associations of California/Union of Health Care Professionals, 2008). These hard work environments due to the nursing shortage in Japan are serious issues that foster ‘weight’ among pregnant nurses. Japanese nurses, in particular, value collectivism and adjust their speaking and behaviour to avoid disrupting the harmony within the team (Omura et al., 2018). Hence, nurses would be concerned about whether their pregnancy disturbs the team, and they try to perform their roles even as they perceive certain limitations.

Feeling inadequate in nursing care is stressful for nurses and related to reduced job satisfaction, burnout and the intention to leave (Stemmer et al., 2022). Frustration with their own professional inability can also be a factor for pregnant nurses to ignore pregnancy symptoms and push on with dangerous work (Quinn, 2016), which can lead to perinatal abnormalities. However, perceiving one's limits is an important process for human development in difficult situations. For example, people with chronic illnesses reconstruct their self‐identity by exploring their personal limitations (Kralik et al., 2004). For nurses, knowing their limitations is considered a crucial skill for providing quality care (Kraus & DuBois, 2017). These findings could explain why the ‘perceive one's limits’ contributes to pregnant nurses' identification of safe ways of working.

Furthermore, joy at work for nurses is an antidote to work‐related stress and is associated with low turnover intention (Carter & Hawkins, 2021). These findings would support the result that ‘delight in nursing’ buffers the influence of ‘weight of one’ and ‘perceive one's limits’ on pregnant nurses and triggers them towards ‘prioritizing the foetus’.

We found that ‘prioritizing the foetus’ differed between multiparous and primiparous participants. The multiparous participants had a strong sense of ‘prioritizing the foetus’ from the beginning of pregnancy and had appropriate management skills. Prior studies demonstrated that the experience of pregnancy and childbirth can lead to significant changes in women's values, self‐efficacy and growth in mental tolerance and flexibility (Brunton et al., 2020) and make their children the top priority (Korukcu, 2020). According to Korukcu (2020), women's ability to cope with problems and self‐management improves with pregnancy and childbirth experience. This finding confirms that the multiparous participants acquired higher management skills based on their previous experiences.

### 5.1 Implications for clinical practice

This study demonstrated the importance of developing ‘prioritizing the foetus’ in the ‘duelling roles’ in the health management of pregnant nurses. Governments and hospitals have significant roles to play in supporting such nurses. At the administrative level, it may be necessary to reconsider nurse staffing systems and also implement policies that easily accommodate the needs of pregnant nurses. Hospitals and nursing managers would need to understand the experiences and difficulties of pregnant nurses and apply maternity policies correctly. Pregnant nurses ought to decipher the process they are following and the importance of ‘prioritizing the foetus’. Primiparas, in particular, could benefit from sharing the health management skills acquired by multiparas.

### 5.2 Strengths and limitations

The strength of this study was the recruitment of pregnant nurses regardless of their residence area, ward, parity or experience with pregnancy term abnormalities. It allowed us to obtain variations regarding the health management of pregnant nurses. The results provide useful insights into health management strategies for pregnant nurses to continue working safely.

The limitation of this study was that the participants were healthy pregnant women who had successfully given birth. Thus, health management among pregnant nurses who have experienced negative pregnancies (e.g., miscarriages or perinatal deaths), complicated pregnancies (e.g., women with diabetes) and older pregnancies may not be consistent with these results. Further research on these high‐risk pregnancy groups may provide a unique perspective on the effective methods of health management for various pregnant nurses.

## 6 CONCLUSIONS

‘Duelling roles’ is a core category describing the process of pregnant nurses' health management. Pregnant nurses feel the ‘weight of one’ as professionals. However, through various interactions, they develop a ‘prioritizing the foetus’ way of working, ‘perceive one's limitations’ and feel ‘delight in nursing’. Sharing and developing the ‘prioritizing the foetus’ mindset and management skills gained by the participants may provide useful suggestions for considering appropriate health management for pregnant nurses. Further research is needed to explore whether this study's results can be extended to nurses experiencing high‐risk pregnancies.

## AUTHOR CONTRIBUTIONS

Study design: Marie Hino, Risa Takashima, Rika Yano. Data collection: Marie Hino. Data analysis: Marie Hino, Risa Takashima, Rika Yano. Manuscript writing: Marie Hino, Risa Takashima, Rika Yano.

## 7 FUNDING INFORMATION

This study was supported by JSPS KAKENHI Grant Number JP 23H03179.

## CONFLICT OF INTEREST STATEMENT

None for each author.

## 8 ETHICS STATEMENT

The ethics committee of the institution to which the authors belonged approved this study (No: 20–54, Date: 06.01.2021). Potential participants were provided verbal and written information about the study’s purpose, data confidentiality, free will participation, the right to withdraw from research participation, the protection of personal information, strict data handling and recording with a voice recorder. All the participants provided written consent. The participants’ anonymity was maintained throughout the study.

## Data Availability

Due to the sensitive nature of the questions asked in this study, survey respondents were assured that raw data would remain confidential and would not be shared.
